# Predisposing Individual Characteristics and Perinatal Outcomes of Women in the Tokyo Metropolitan Area Who Initiate Prenatal Care Late in Their Pregnancy: A Case-Control Study

**DOI:** 10.5402/2012/945628

**Published:** 2012-08-08

**Authors:** Jun Kakogawa, Miyuki Sadatsuki, Takeji Matsushita, Takuro Simbo

**Affiliations:** ^1^Department of Obstetrics and Gynecology, National Center for Global Health and Medicine, Tokyo 162-8655, Japan; ^2^Department of Pediatrics, National Center for Global Health and Medicine, Tokyo 162-8655, Japan; ^3^Department of Clinical Research and Informatics, National Center for Global Health and Medicine, Tokyo 162-8655, Japan

## Abstract

*Purpose*. The purpose of this study was to investigate the individual characteristics and perinatal outcomes of women who initiate prenatal care late in their pregnancy in the Tokyo metropolitan area. *Methods*. Retrospective study. The study enrolled all women at our hospital who initiated prenatal care after 22 weeks of gestation (late attenders) and control women who initiated prenatal care prior to 11 weeks of gestation participated in the study at the National Center for Global Health and Medicine between January 1, 2007 and June 30, 2011. We compared the maternal characteristics and perinatal outcomes of late attenders with those of the control group. *Results*. A total of 121 late attenders and 1,787 controls were enrolled. Late attenders had a higher incidence of unmarried compared with the control group (*P* < 0.01). There were no differences in the incidence of preterm delivery and low birth weight; however, babies of the late attenders had a higher incidence of admission to the neonatal intensive care unit compared with the control group (*P* < 0.01). *Conclusions*. Our results indicate that there is a pressing need for further steps to promote the importance of receiving prenatal care during pregnancy.

## 1. Introduction

Prenatal care (PNC) is a widely used preventive health service, and it is recognized as being important for the health of pregnant women and their babies. There is an international consensus that the initiation of PNC visits should start during the first trimester of pregnancy [1]. The early initiation of PNC and regular follow-ups are essential to enable the identification and reduction of factors related to maternal morbidity and their neonatal consequences. Previous studies have reported that the late initiation of PNC is associated with low income [2], unmarried status of the mother [3], parity [4], young maternal age [4, 5], unintended pregnancy [3, 6], and a lack of child care [7]. The Japan Society of Obstetrics and Gynecology (JSOG) [8] recommends approximately 14 PNC visits, with the first visit held prior to 11 weeks of gestation. In Japan, the financial support from public funds for 14 PNC visits is currently provided to all pregnant women. Perinatal assistance in the metropolitan area of Tokyo is characterized by easily accessible and high-quality maternity care. However, our previous work has demonstrated that the socioeconomic and demographic barriers to adequate PNC attendance still exist in the metropolitan area of Tokyo [9].

Socioeconomic problems can affect children, even before birth. A low socioeconomic status during pregnancy is a risk factor for adverse pregnancy outcomes, such as premature birth and low birth weight; low socioeconomic status is strongly associated with morbidity and mortality [10, 11]. Furthermore, child abuse is more common among families with low socioeconomic status [12]. The consequences of child abuse are significant; for example, abused children are more likely to have greater psychosocial problems, low self-esteem, and morbidity [13]. In the United States, an intervention initiative to prevent child abuse was developed by David Olds that focuses on high-risk families, called the Nurse Family Partnership (NFP) [14]. Currently in Japan, child abuse is also a serious social issue. In 2009, approximately 40 percent of the children in Japan who died were less than 1 year old [15]. Although there is an urgent need to establish early interventions to prevent future child abuse in high-risk pregnant women, there is also a notable lack of interventions that systematically address risk factors for potential child abuse during the prenatal period in Japan.

The burden of unintended pregnancies is still a serious public health issue worldwide [16]. In Japan, overlapping risk factors for unintended pregnancies and child abuse are currently a primary focus of concern. Induced abortion after 22 weeks of gestation is prohibited by law in Japan. The late initiation of PNC, especially when the first visit is initiated after 22 weeks of gestation, may possibly indicate that a woman did not receive PNC because her pregnancy was unintended. Thus, the purpose of this study, which was conducted at the National Center for Global Health and Medicine (NCGM) in the metropolitan area of Tokyo, was to investigate the individual characteristics and perinatal outcomes of women who initiated PNC after 22 weeks of gestation.

## 2. Materials and Methods

The National Center for Global Health and Medicine (NCGM) is the only public hospital in Shinjuku (the metropolitan area of Tokyo) equipped with a neonatal intensive care unit (NICU), wherein medical care is available to the relatively deprived population of Shinjuku and the surrounding areas. Most women with low socioeconomic status who reside in this area deliver their infants at the NCGM. The study enrolled all women with singleton pregnancies who initiated PNC after 22 weeks of gestation at the NCGM between January 1, 2007 and June 30, 2011 (i.e., “late attenders”). The control group included all women who initiated PNC prior to 11 weeks of gestation at the NCGM during the study period. These control individuals did not have any medical problems that inherently required additional PNC (e.g., heart disease, hypertension, or renal disease). The exclusion criterion in both groups was multiple pregnancies. The study was approved by the institutional review board of the NCGM.

The data were retrospectively retrieved from a perinatal database, followed by an individual chart review. The outcomes that we investigated included the following maternal characteristics: maternal age, marital status, relationship with the child's father, cigarette smoking and alcohol consumption during pregnancy, obstetric history, obstetric outcomes, and neonatal morbidity. We compared the maternal characteristics and perinatal outcomes of the late attenders with those of the control group.

The following definitions were used in this study. Young maternal age was defined as less than 20 years of age, and maternal age was divided into four categories: less than 20, 20–29, 30–39, and over 40 years of age. Unmarried status was defined as any civilian status, other than marriage, and included cohabiting, single, widowed, and divorced women. Cigarette smoking and alcohol consumption during pregnancy were recorded in a perinatal database as a yes/no response. The gestational age was estimated based on the first day of the last menstrual period. The diagnosis of threatened preterm delivery (TPD) was based on clinical evidence of painful uterine contractions and cervical dilatation. Pregnancy-induced hypertension (PIH) was defined as a blood pressure reading of 140/90 mmHg or higher after the 20th week of gestation. The diagnosis of gestational diabetes mellitus (GDM) was determined by a 75 g oral glucose tolerance test, with plasma glucose measurements taken after fasting at 1 and 2 h. At least two of the following plasma glucose values were exceeded in women who screened positive for GDM: 100 mg/dL (fasting), 180 mg/dL (1 h), and 150 mg/dL (2 h). We defined preterm delivery as a gestational age of less than 37 weeks. Low birth weight (LBW) was defined as a weight less than 2,500 g, whereas a birth weight greater than 4,000 g was defined as macrosomia. A stillbirth was defined as an intrauterine fetal death (IUFD) at 22 or more weeks of gestation. Early neonatal death was defined as the death of a neonate during the first seven days of life, whereas a late neonatal death was defined as death between 8 and 28 days after birth. The admission rate to the neonatal intensive care unit (NICU) was recorded based on the infants who required more than 24 hours of surveillance.

The data are presented as the means ± SD. The Statistical Package for the Social Sciences (SPSS, version 20.0 for Windows; SPSS, Inc., Chicago, IL) was used to analyze the data. The dichotomous data were compared using Chi-square tests, and Fisher's exact test was applied when the minimal-estimated expected value was less than five. The continuous variables were analyzed with a Student's *t*-test. A *P*-value of < 0.05 was considered statistically significant.

## 3. Results

A total of 121 late attenders and 1,787 controls were enrolled in this study. There were 2,221 deliveries at the NCGM during the study period. The proportion of late attenders (5.4%) among all deliveries was small during the study period.  Table 1 describes the demographic and maternal characteristics of the study participants. In the late attenders group, the mean maternal age was less than that in the control group (*P* < 0.01). The late attenders were found to have a higher incidence of young maternal age compared with the control group (*P* < 0.01). Although there were no differences in primiparas and multiparas between the late attenders and the controls (*P* = 0.39), the control group had lower incidences of multiparas with 4 or more previous deliveries (*P* < 0.01) and induced abortion (*P* < 0.01) compared to the late attenders. The late attenders were more likely to have an unmarried status, a history of divorce, no relationship with the child's father, and a history of cigarette smoking and/or alcohol consumption compared to the control group (*P* < 0.01).

Table 2 describes the maternal complications across the study groups. There were no differences in the incidences of TPD, PIH, GDM, placental abruption, placenta previa, and preterm premature rupture of membrane (pPROM) or IUFD between the late attenders and the controls. With regard to infections, the late attenders were found to have a higher incidence of *Chlamydia trachomatis* infection (*P* < 0.01), syphilis (*P* < 0.01), hepatitis B (*P* < 0.01), and hepatitis C (*P* < 0.01) compared with the control group; however, there were no differences in the incidence of human immunodeficiency virus (HIV) between the two groups.

Table 3 describes the pregnancy outcomes and neonatal characteristics in the study groups. There were no maternal or neonatal deaths in either group. No differences were observed in the mean gestational age at maternal delivery, the incidence of stillbirth, preterm delivery, forceps delivery, or cesarean section between late attenders and controls. Although there were no group differences in the incidence of low birth weight, macrosomia, and low Apgar scores, the mean birth weight in the late-attender group was lower than that in the control group (*P* = 0.03). Babies born to late attenders were also found to have a higher incidence of admission to the NICU compared with babies born to members of the control group (*P* < 0.01).

Regarding the reasons for admission to the NICU, the neonates of the late attenders were found to have a higher incidence of neonatal infection (*n* = 8 versus *n* = 15; *P* < 0.01) and respiratory distress (*n* = 11 versus *n* = 13; *P* < 0.01), although there were no group differences in the causes of preterm delivery, LBW, hyperbilirubinemia, or asphyxia. Congenital syphilis was not identified in members of both study groups. Although all mothers with HIV infection had been treated with anti-viral therapy, received elective cesarean section, and avoided breastfeeding to prevent maternal-to-child infection, only one neonate in the late attender group was diagnosed with HIV infection at birth. The mother of this child had initiated treatment for HIV infection at 34 weeks of gestation due to the late initiation of PNC. The late attenders were found to have a higher incidence of admission to the orphanage compared to rates in the control group (*n* = 21 versus *n* = 5; *P* < 0.01).

## 4. Discussion

The early initiation of PNC is critically important to ensure the receipt of early medical follow-ups and timely information regarding screening tests. We found that 5.4% of women in the metropolitan area of Tokyo initiated PNC after 22 weeks of gestation. It has been reported that the rates of women who initiate PNC after 12 weeks of gestation were 10.8% in the Brussels metropolitan region in Belgium [17] and 13.4% in the Arajuca metropolitan region in Brazil [18]. Previous studies have reported that the late initiation of PNC is associated with maternal age [4, 5], smoking status [5], multiparity [4], unmarried status of the mother [3], young maternal age [4, 5], and lack of child care [7]. As we have described, women who initiate PNC after 22 weeks of gestation in the metropolitan area of Tokyo exhibit similar demographic characteristics. Unplanned and unexpected pregnancies have also been reported as being predictive for the late initiation of PNC [6]. In the present study, the incidence of unintended pregnancy in the late attenders was inferred to be elevated in comparison with that of the controls because the late attenders' neonates had a higher incidence of admission to the orphanage compared with the control rates. However, we did not directly measure the incidence of unintended pregnancy. The association between income and the initiation of care during pregnancy has also been found in the Brussels metropolitan region [17]. Although we did not directly examine income level or occupational status in the present study, the late attenders had a higher incidence of young maternal age, unmarried status, and the absence of a relationship with the child's father. Therefore, it is likely that the late attenders had low socioeconomic status.

Several studies have reported that inadequate PNC carries a substantially elevated risk of severe adverse prenatal outcomes, such as preterm birth [19], placental abruption [20], low birth weight [19, 20], stillbirth [21], and neonatal death [19]. Our previous report showed that women who did not receive PNC had a 22.2% incidence of preterm birth and a 28.8% incidence of low birth weight [9]. Interestingly, in the present study, the incidence of preterm birth was 6.6%, and the incidence of low birth weight was 9.0% in late attenders. Furthermore, we found no differences in obstetrical complications, such as preterm birth, mode of delivery, and asphyxia, in either group. One explanation for the lack of differences in perinatal outcomes between these populations is that the late attender group was able to receive PNC and be provided with high-quality care despite their initiation of PNC after 22 weeks of gestation. Another possible explanation is that the financial support for PNC from public funds contributed to regular PNC attendance and provision of the appropriate interventions in the late attender group.

In the present study, we found a reduced mean birth weight in the late attenders compared to the controls; however, there was no difference in the incidence of LBW. An unborn child is at risk for adverse pregnancy outcomes depending on the negative health patterns of the mother. Cigarette smoking [11] and alcohol consumption [22] during pregnancy are associated with low birth weight. The present study showed that the late attenders had a higher incidence of cigarette smoking and alcohol consumption than the control group. Thus, one explanation of the elevated incidence of lower-mean birth weight in the late attender group is that cigarette smoking and alcohol consumption during pregnancy affected the mean birth weight of children born to these mothers. Furthermore, teenagers have an increased risk of delivering small-for-gestational-age (SGA) infants [23]. Another explanation for the higher incidence of the reduced mean birth weight in the late attenders is that the late attenders were more likely to be teenagers.

The present study showed that the late attenders had a higher incidence of *Chlamydia trachomatis *infection, syphilis, hepatitis B, hepatitis C, and a history of induced abortion than the control group. Wenman et al. [24] reported that the incidence of *Chlamydia trachomatis* among pregnant women was less than 1%. In the present study, we found a 15.7% incidence of *Chlamydia trachomatis *infections. The late attenders had approximately three times the incidence of a history of induced abortion compared to the controls in the present study. This result suggests that a lack of awareness regarding safe health behaviors may affect the late initiation of PNC. A higher incidence of sexually transmitted infections (STIs) was associated with a higher incidence of admission to the NICU and neonatal infection in the late attenders compared with the controls. We did not examine the incidence of intimate partner violence in the present study. Women who experience intimate partner violence are at risk for STIs [25]. Additional studies are needed to investigate whether intimate partner violence could affect the initiation of PNC or the incidence of STIs in the metropolitan area of Tokyo.

The predisposing demographic characteristics of women who initiated PNC after 22 weeks of gestation were described in this study. Considering that no differences were found in perinatal outcomes, such as LBW, it has been suggested that policy makers focus on issues that are relevant after the initiation of PNC, such as the provision of high-quality care and financial support. Downe et al. [6] reported that a lack of information regarding how to access care or a lack of awareness regarding the range of available services are barriers to prenatal care for marginalized women in high-income countries. Our results suggest that as a next step, healthcare policy makers should focus on establishing informational strategies. These strategies should be aimed at raising the awareness of PNC, providing access to PNC during pregnancy, and shifting the focus from a generalized to an individualized approach to care for women with risk factors identified in this study. The late initiation of PNC in women with socioeconomic problems may result in unfavorable infant health outcomes. In Japan, there is no established health care system like the NFP, which has been utilized to prevent child abuse in high-risk families. Given that child abuse is a serious social problem, it seems that our results could be utilized to help establish a prenatal intervention initiative to prevent child abuse.

Some limitations of our study need to be addressed. First, this study was conducted in a single center. However, we believe that the results of the present study are compatible with the demographic characteristics of women who initiate PNC after 22 weeks of gestation in the metropolitan area of Tokyo because the NCGM is the only public hospital equipped with a NICU, and medical care is made available to populations in the surrounding area with socioeconomic problems as well as to most pregnant women. Second, our case control analyses were performed by defining the groups based on when they initiated PNC, either after 22 or more weeks of gestation or prior to 11 weeks of gestation. Therefore, our study did not include women who initiated PNC between 12 and 21 weeks of gestation. Furthermore, our study was not designed to evaluate women who did not receive any form of PNC, and the determinants of late PNC initiation are likely to differ from factors related to the absence of PNC. Further investigations should be focused on analyzing the differences in factors related to the late initiation of PNC and the absence of PNC. Third, our study did not assess the reasons for the late initiation of PNC. It is possible that the consequences of the predisposing variables on the late initiation of PNC are more complicated than the study revealed. Therefore, further studies should focus on the analysis of other demographic factors, such as economic status and educational levels. Finally, our analysis was limited to singleton pregnancies; therefore, multiple-gestational pregnancies were not represented.

In conclusion, the present study elucidated the demographic characteristics and perinatal outcomes of women who initiated PNC after 22 weeks of gestation in the metropolitan area of Tokyo. Our findings confirmed that the maternal demographic characteristics are important factors associated with the late initiation of PNC in this area. This study emphasizes new insights into the reduction of the incidence of obstetrical complications, such as LBW and preterm birth, by utilizing the financial support provided by public funds and providing high-quality care. However, our results indicate that the fetuses of mothers who initiate PNC after 22 weeks of gestation are more likely to be exposed to infection, tobacco and alcohol. Our results indicate that there is a pressing need for the development of further steps to promote the importance of receiving PNC during pregnancy. Further investigations are needed to enable the development of effective strategies for health education and to increase the dissemination of information regarding PNC.

## Figures and Tables

**Table 1:** Demographic and maternal characteristics of the study groups.  The table indicates the number of women in each group and the data are presented as the mean ± standard deviation.  In the late attenders group, the mean maternal age was less than that in the control group (*P* < 0.01).  Late attenders had statistically significant higher rates of unmarried status, history of divorce, absence of a relationship with the child's father, cigarette smoking and alcohol consumption, and history of abortion compared with those of the control group (*P* < 0.01).

	Late attenders (*n* = 121)	Control group (*n* = 1,787)	*P* value
Maternal age (years), mean ± SD	26.4 ± 6.6	32.1 ± 5.1	<0.01
<20 years	26 (21.5%)	17 (1.0%)	<0.01
20 to 29 years	57 (47.1%)	501 (28.0%)	
30 to 39 years	37 (30.6%)	1150 (64.4%)	
≥40 years	1 (0.8%)	119 (6.7%)	
Primiparity/multiparity	65/56	1,034/753	0.39
Multiparas with 4 or more previous deliveries	7 (5.8%)	19 (1.1%)	<0.01
Women who had a history of induced abortion	53 (43.8%)	263 (14.7%)	<0.01
Unmarried	102 (84.3%)	52 (2.8%)	<0.01
Women who had a history of divorce	43 (35.5%)	49 (2.7%)	<0.01
Women who had no relationship with the child's father	84 (69.4%)	36 (2.0%)	<0.01
Cigarette smoking	53 (43.8%)	58 (3.2%)	<0.01
Alcohol consumption	36 (29.8%)	54 (3.0%)	<0.01

**Table 2:** Maternal complications in the study groups.  The table indicates the number of women in each group.  Late attenders had a higher incidence of *Chlamydia trachomatis* infection, syphilis,  hepatitis B, and hepatitis C compared with the control group (*P* < 0.01).

	Late attenders (*n* = 121)	Control group (*n* = 1,787)	*P* value
Threatened preterm delivery	3 (2.5%)	83 (4.6%)	0.36
Pregnancy-induced hypertension	2 (1.7%)	30 (1.7%)	0.16
Gestational diabetes mellitus	1 (0.8%)	17 (1.0%)	1.00
Placental abruption	0 (0%)	7 (0.4%)	1.00
Placenta previa	2 (1.7%)	7 (0.4%)	0.10
Preterm premature rupture of membrane	1 (0.8%)	17 (1.0%)	1.00
Intrauterine fetal death	1 (0.8%)	7 (0.4%)	0.40
Infection			
*Chlamydia trachomatis *	19 (15.7%)	6 (0.3%)	<0.01
Syphilis	5 (4.1%)	2 (0.1%)	<0.01
Hepatitis B virus	4 (3.3%)	7 (0.4%)	<0.01
Hepatitis C virus	6 (5.0%)	15 (0.8%)	<0.01
Human immunodeficiency virus	3 (2.5%)	15 (0.8%)	0.10

**Table 3:** Pregnancy outcomes and neonatal characteristics in the study groups.  The table indicates the number of women in each group and the data are presented as the mean ± standard deviation. The mean birth weight in the late-attender group was reduced compared to that in the control group (*P* = 0.03). Babies born to the late attenders were found to have a higher incidence of admission to the NICU compared with babies born to the control group (*P* < 0.01).

	Late attenders (*n* = 121)	Control group (*n* = 1,787)	*P* value
Gestational age at delivery (weeks), mean ± SD	38.9 ± 1.9	38.9 ± 1.6	0.93
Preterm birth	8 (6.6%)	105 (5.9%)	0.69
Still birth	1 (0.8%)	7 (0.4%)	0.40
Mode of delivery			
Spontaneous delivery	96 (79.3%)	1412 (79.0%)	1.00
Forceps delivery	8 (6.6%)	89 (5.0%)	0.39
Cesarean section	17 (14.1%)	286 (16.0%)	0.69
Birth weight (g), mean ± SD	2,934.3 ± 383.9	3,019.3 ± 440.0	0.03
Low birth weight (<2,500 g)	11 (9.1%)	154 (8.6%)	0.86
Macrosomia (≥4,000 g)	1 (0.8%)	24 (1.3%)	1.00
Low Apgar score (<7) 1 min	7 (5.8%)	72 (4.0%)	0.34
Low Apgar score (<7) 5 min	1 (0.8%)	17 (1.0%)	1.00
Admission to neonatal intensive care unit	23 (19.0%)	149 (8.3%)	<0.01

## Abbreviations

PNC: Prenatal care
NFP: Nurse family partnership
LBW: Low birth weight
TPD: Threatened preterm delivery
PIH: Pregnancy-induced hypertension
GDM: Gestational diabetes mellitus
HIV: Human immunodeficiency virus.
